# Combined Metabolite and Transcriptome Profiling Reveals the Norisoprenoid Responses in Grape Berries to Abscisic Acid and Synthetic Auxin

**DOI:** 10.3390/ijms22031420

**Published:** 2021-01-31

**Authors:** Lei He, Nan Meng, Simone D. Castellarin, Yu Wang, Qi Sun, Xiang-Yi Li, Zhi-Gang Dong, Xiao-Ping Tang, Chang-Qing Duan, Qiu-Hong Pan

**Affiliations:** 1 Center for Viticulture & Enology, College of Food Science and Nutritional Engineering, China Agricultural University, Beijing 100083, China; helei@cau.edn.cn (L.H.); mn@cau.edu.cn (N.M.); wangyu_0919@cau.edu.cn (Y.W.); sq18813017889@cau.edu.cn (Q.S.); vale_li@126.com (X.-Y.L.); chqduan@cau.edu.cn (C.-Q.D.); 2 Key Laboratory of Viticulture and Enology, Ministry of Agriculture and Rural Affairs, Beijing 100083, China; 3 Wine Research Centre, Faculty of Land and Food Systems, The University of British Columbia, Vancouver, BC V6T 1Z4, Canada; sdcastel@mail.ubc.ca; 4 Institute of Pomology, Shanxi Academy of Agricultural Sciences, Taiyuan 030801, China; gssdzg@163.com (Z.-G.D.); txp-19590401@163.com (X.-P.T.)

**Keywords:** norisoprenoid, grape berry ripening, abscisic acid, naphthaleneacetic acid, gene expression, DAP-seq, alternative splicing

## Abstract

The abscisic acid (ABA) increase and auxin decline are both indicators of ripening initiation in grape berry, and norisoprenoid accumulation also starts at around the onset of ripening. However, the relationship between ABA, auxin, and norisoprenoids remains largely unknown, especially at the transcriptome level. To investigate the transcriptional and posttranscriptional regulation of the ABA and synthetic auxin 1-naphthaleneacetic acid (NAA) on norisoprenoid production, we performed time-series GC-MS and RNA-seq analyses on *Vitis vinifera* L. cv. Cabernet Sauvignon grape berries from pre-veraison to ripening. Higher levels of free norisoprenoids were found in ABA-treated mature berries in two consecutive seasons, and both free and total norisoprenoids were significantly increased by NAA in one season. The expression pattern of known norisoprenoid-associated genes in all samples and the up-regulation of specific alternative splicing isoforms of *VviDXS* and *VviCRTISO* in NAA-treated berries were predicted to contribute to the norisoprenoid accumulation in ABA and NAA-treated berries. Combined weighted gene co-expression network analysis (WGCNA) and DNA affinity purification sequencing (DAP-seq) analysis suggested that VviGATA26, and the previously identified switch genes of myb RADIALIS (VIT_207s0005g02730) and MAD-box (VIT_213s0158g00100) could be potential regulators of norisoprenoid accumulation. The positive effects of ABA on free norisoprenoids and NAA on total norisoprenoid accumulation were revealed in the commercially ripening berries. Since the endogenous ABA and auxin are sensitive to environmental factors, this finding provides new insights to develop viticultural practices for managing norisoprenoids in vineyards in response to changing climates.

## 1. Introduction

Norisoprenoids are among the most important grape-derived flavor compounds in wine, especially for non-Muscat cultivars. With very low olfactory perception thresholds and powerful aroma properties, they contribute to the floral and fruity attributes of grapes and wines [1,2]. Due to the important sensory contribution of norisoprenoids, extensive researches were conducted on the response of these compounds to some treatments such as synthetic auxin application [3], sunlight exposure [4], and partial rootzone drying [5]. Norisoprenoids are carbonyl compounds with 9, 10, 11, or 13 carbon atoms which derived from oxidative degradation of carotenoids, a diverse group of C40 pigments in plant [6]. Previous studies have identified three carotenoid cleavage dioxygenase (CCD) enzyme family members (CCD1, CCD4a, and CCD4b) that can catalyze the cleavage of carotenoid substrates, and then produce norisoprenoids [6,7]. The expression of these three genes has been found to significantly increase in grape berries at the onset of ripening (veraison), compared to pre-veraison berries [7]. Chen et al. observed that *VviCCD1* increased from the green stage and peaked at around veraison, while the transcript abundance of *VviCCD4b* and *VviCCD4a* started to increase from veraison and after veraison, respectively [8]. They suggested that the higher level of norisoprenoids content in response to distinct climate was related to up-regulated *VviCCD4b*. However, the other regulatory mechanisms of norisoprenoid accumulation like alternative splicing, transcriptional regulation by transcription factors (TFs), and regulatory network with other genes are poorly understood. Alternative splicing (AS), as one important posttranscriptional regulatory mechanism that can affect mRNA stability and increase protein diversity, has recently gained attention in grapevine research. Vitulo et al. found that 30% (8668) of the grapevine predicted genes undergo AS with 64% of these alternatively spliced genes possess more than two isoforms and produce 32,395 different isoforms in grape berry [9]. They also suggested that AS can affect miRNA target sites, indicating its contribution to the transcriptional complexity and regulation. Furthermore, it was found that AS seems to be conserved among different varieties [10]. Using combined transcriptomic and proteomic analysis, Jiang and the colleagues have proven that AS plays an important posttranscriptional regulatory role in the response of grape leaves to high-temperature stimuli [11]. In the aspect of transcriptional regulation, we recently have identified a MADs family transcription factor (TF) *VviMADS4* directly down-regulating *VviCCD4b* expression [12], and a *VviWRKY40* transcription factor responsible for monoterpenoid glycosylation [13]. However, to date, there is no report uncovering the potential TFs positively regulating the biosynthesis of norisoprenoids and other volatile organic compounds in grape berry.

The norisoprenoids markedly increase at around veraison [8,14], which is a crucial shift point for grape berries to change from green/immature to ripe/mature, and encompasses physiological and metabolic changes. These changes include berry softening, sugar, anthocyanin, and flavor accumulation, and parallel an increase of abscisic acid (ABA) level and a decrease of auxin level [15,16,17]. Hormones are proven to play a major role in controlling several ripening-associated processes like fruit coloration and aroma development [18,19]. Among the hormones accumulated in grape berry, ABA and auxin are considered critical for the regulation of ripening progression. Several studies confirmed that ABA increase and auxin decline are tightly associated with the initiation of ripening [17,18,20]. However, the understanding of the role of the two hormones in regulating norisoprenoid production is limited. One report mentioned that the exogenous application of auxin-like compounds can delay ripening and simultaneously affect the concentration of norisoprenoids in wines [3]. Moreover, our previous study preliminarily investigated the effects of ABA and synthetic auxin 1-naphthaleneacetic acid (NAA) on anthocyanins and volatile compounds in grape berry at the beginning of ripening [21]. In the present study, we further evaluated the roles of ABA and NAA in regulating the biosynthesis of norisoprenoid at both the metabolite and transcriptional levels. The purpose of this work was to elucidate the regulatory mechanism underlying the effects of ABA and NAA application on the accumulation of norisoprenoids, a class of important carotenoid degradation products in grape berry, and to find the potential regulatory genes. The gained results will provide new insight into controlling metabolism from carotenoids to norisoprenoids. Due to the important contribution of norisoprenoid compounds to the varietal aroma of neutral variety wines like Cabernet Sauvignon, the research outcome also can guide viticulturists on the decision of viticulture management that leads to a good varietal aroma.

## 2. Results

### 2.1. Effects of ABA and NAA on Berry Development and Ripening

#### 2.1.1. Evolution of Sugar and Acidity

**Figure 1:**
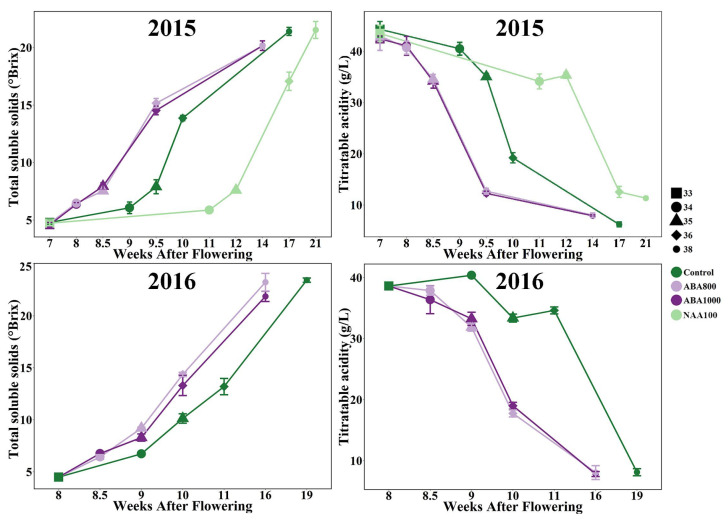
Total soluble solids and titratable acidity in two consecutive seasons. The different shape represents the phenological stage: square (E-L 33 stage), big circle (E-L 34 stage), triangle (E-L 35 stage), rhombus (E-L 36 stage), small circle (E-L 38 stage). The lines of green, light purple, purple and light green represent Control, 800 mg/L abscisic acid (ABA) (ABA800), 1000 mg/L ABA (ABA1000), and 100 mg/L synthetic auxin 1-naphthaleneacetic acid (NAA) (NAA100), respectively. Bars represent ± standard deviation.

Two ripening parameters of grape berry, total soluble solids (TSS) and titratable acidity (TA), were compared between the treatments and the control at five phenological stages. There was no significant difference in TSS and TA at E-L 33 stage before the treatments were conducted. After ABA spraying in 2015 and 2016, the initiation of ripening (E-L 35 stage) of grape berries was advanced about one week and the subsequent ripening process was also accelerated by both ABA800 and ABA1000 treatments (Figure 1). Both ABA1000 and ABA800-treated berries accumulated TSS faster than the control. As a result, these berries achieved technological maturity (E-L 38 stage) about 17 days ahead of the control in 2015 and 25 days earlier than the control in 2016 (Figure 1). In contrast, NAA-treated berries reached technological maturity about 25 days later than the control group owing to the slower speed of TSS accumulation. At the same time, the ABA application markedly promoted the TA decrease, whereas NAA suppressed it. It was interesting to see that the influence of NAA mainly laid in delaying the onset of grape berry ripening (from E-L33 to E-L 35) and extending the berry coloration process (from E-L35 to E-L 36)

#### 2.1.2. Changes of Endogenous ABA and Auxin Biosynthesis and Signaling Pathway

**Figure 2:**
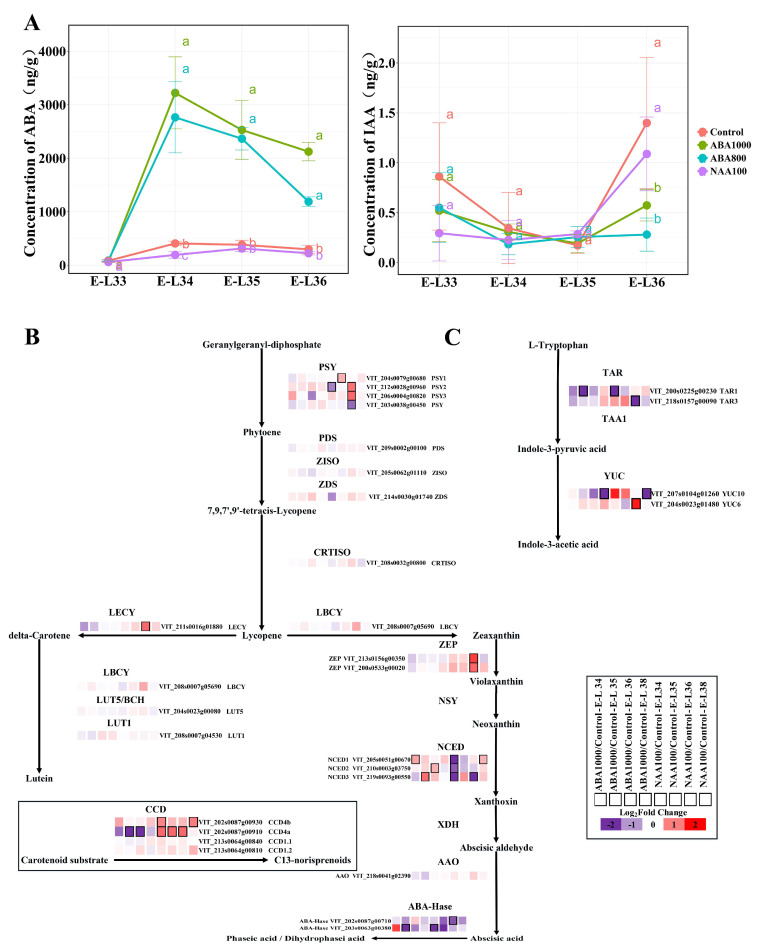
Effects of ABA and NAA on hormone accumulation and related gene expression during berry development and ripening. (**A**) Concentrations of endogenous ABA and indole-3-acetic acid protein (IAA) from E-L 33 to E-L 36 stage. Bars represent ± SE and different letters indicate significant differences (*p* = 0.05). (**B**) Pathways analysis of genes involved in ABA and norisoprenoid biosynthesis. Purple and red boxes indicate downregulated and upregulated genes, the colors of the boxes represent the intensity of the expression fold changes (log2). Genes with significant expression changes compared with the control groups in each developmental stage are indicated by bold margins. PSY, phytoene synthase; PDS, 15-cis-phytoene desaturase; ZISO, zeta-carotene isomerase; ZDS, zeta-carotene desaturase; CRTISO, prolycopene isomerase; LECY, lycopene epsilon-cyclase; LBCY, lycopene beta-cyclase; LUT5, beta-ring hydroxylase; BCH, beta-carotene 3-hydroxylase; LUT1, carotene epsilon-monooxygenase; ZEP, zeaxanthin epoxidase; NSY, neoxanthin synthase; NCED, 9-cis-epoxycarotenoid dioxygenase; XDH, xanthoxin dehydrogenase; AAO, abscisic-aldehyde oxidase; CCD, carotenoid cleavage dioxygenase. (**C**) Pathways analysis of genes involved in auxin biosynthesis. TAR, tryptophan aminotransferase related 1; TAA1, tryptophan aminotransferase arabidopsis1; YUC, YUC flavin monooxygenase.

We then investigated the responses of endogenous ABA and auxin biosynthesis and signaling to ABA and NAA spraying. The levels of endogenous ABA and indole-3-acetic acid protein (IAA) in grape berries were measured at stages of E-L 33, E-L 34, E-L 35, and E-L 36 (Figure 2A). There was no significant difference in ABA and IAA concentration among all samples at E-L 33, before treatment application. The ABA concentration peaked at E-L 34 stage then decreased until ripening in the control group. In contrast, the concentration of ABA increased sharply at E-L 34 after ABA1000 and ABA800 application, while the ABA accumulation in NAA-treated samples was inhibited by NAA treatment. Both ABA treated and control samples were found to have their ABA peak at E-L 34, whereas the peak time was delayed to E-L 35 stage in NAA treated samples. Transcriptomic data of ABA1000, NAA100, and the control in 2015 showed that three VviNCEDs, which are involved in both norisoprenoid and ABA biosynthesis, were found to be up-regulated by ABA treatments at three different developmental stages, respectively (Figure 2B). On the contrary, the VviNCEDs were down-regulated by NAA treatment, especially at E-L 34 stage. It can explain the higher concentrations of ABA in ABA-treated berries while lower ABA levels in NAA-treated berries. Additionally, different from ABA, the concentration of IAA was very low in all samples. There was a decline of the IAA concentration at E-L 34 and then a small increase was found at the following stages. Generally, no differences were observed among treatments and control; however, a lower amount of IAA was found in ABA samples at E-L 36. We observed that ABA application suppressed the expression of the genes encoding tryptophan aminotransferase related 1 (TAR1, VIT_200s0225g00230) and YUC flavin monooxygenase 10 (YUC10, VIT_207s0104g01260) at E-L 35 and E-L38 stages, respectively (Figure 2C). The VviTAR1, VviTAR3, and VviYUC10 also showed low levels in NAA-treated berries at E-L 34, E-L 36, and E-L38 stages, respectively. The VviYUC6 (VIT_204s0023g01480) was markedly up-regulated by NAA treatment at E-L 36 stage.

**Figure 3:**
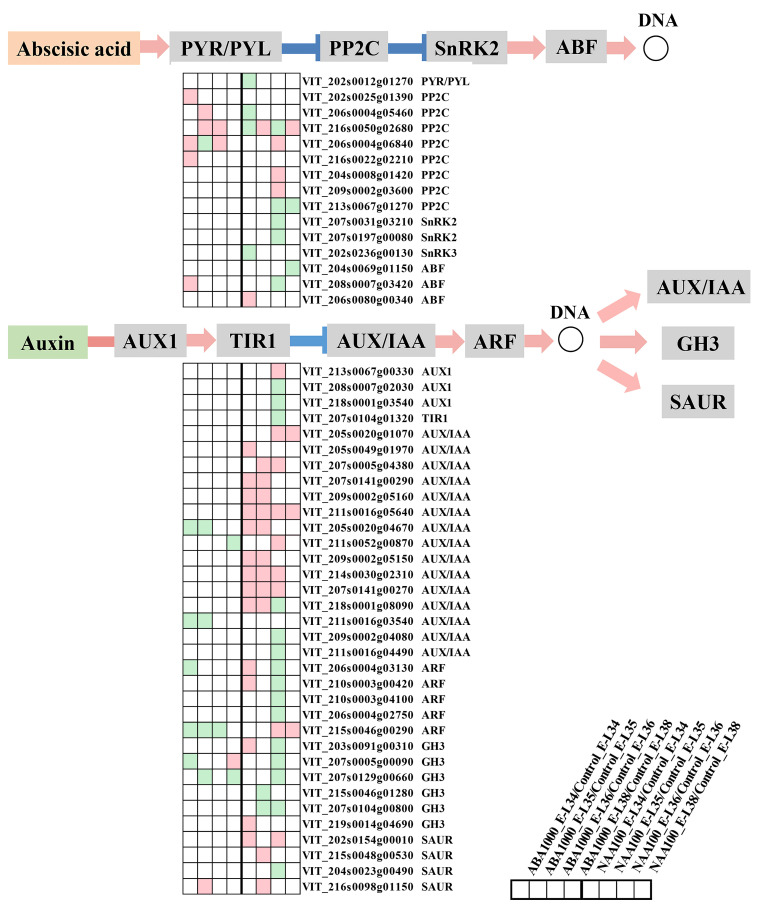
Expression of genes involved in ABA and auxin signaling pathway. Green and cyan boxes represent significantly downregulated and upregulated genes, respectively, in treated berries compared to the control.

A heatmap displaying the regulation of ABA and auxin signal transduction pathway by exogenous ABA and NAA is reported in Figure 3. In the ABA signaling pathway, the binding of ABA with the PYR/PYL receptor inhibits the protein serine/threonine phosphatase 2C (PP2C), which inhibits the kinase SnRK2 by its phosphatase activity [22]. The SnRK2s released from the PP2Cs inhibition can activate the downstream ABA-responsive element binding factors (ABFs), leading to the activation of ABA-responsive genes [23]. In ABA-treated berries, the expression of ABA receptor gene VviPYR/PYL was unchanged in comparison to the control, and one VviABF (VIT_208s0007g03420) was up-regulated at E-L 34 stage. Unexpectedly, the transcript abundance of five VviPP2Cs (VIT_202s0025g01390, VIT_206s0004g05460, VIT_216s0050g02680, VIT_206s0004g06840, and VIT_216s0022g02210) was significantly increased at E-L 34, E-L 35, or E-L 36 stages (Figure 3). In contrast, NAA application significantly decreased the expression of one VviPYR/PYL (VIT_202s0012g01270), two VviPP2Cs (VIT_206s0004g05460 and VIT_216s0050g02680), and one VviSnRK2 (VIT_202s0236g00130) at E-L 34 stage, as well as one VviPP2C (VIT_213s0067g01270), two VviSnRK2s (VIT_207s0031g03210 and VIT_207s0197g00080), and two VviABFs (VIT_204s0069g01150 and VIT_208s0007g03420) at E-L 36 or 38 stage. This seems to indicate that NAA application down-regulated ABA signaling activity. Regarding auxin signaling, we investigated the expression of genes encoding auxin influx carrier (AUX1), transport inhibitor response 1 (TIR1), auxin/indole-3-acetic acid protein (AUX/IAA), auxin response factor (ARF), indole-3-acetic acid-amido synthetase (GH3) and small auxin-up RNA (SAUR) [24]. ABA markedly down-regulated the expression of auxin signaling genes including four VviAUX/IAAs (VIT_205s0020g04670, VIT_215s0046g00290, VIT_211s0052g00870, and VIT_211s0016g03540), two VviARFs (VIT_206s0004g03130 and VIT_215s0046g00290), and two VviGH3s (VIT_207s0005g00090 and VIT_207s0129g00660) at the early stage of E-L 34 or E-L 35. On the contrary, NAA treatment up-regulated the expression of most VviAUX/IAAs, two VviARFs (VIT_206s0004g03130 and VIT_210s0003g00420), two VviGH3s ((VIT_203s0091g00310 and VIT_219s0014g04690), and of two VviSAURs (VIT_202s0154g00010 and VIT_215s0048g00530). One VviSAUR (VIT_216s0098g01150) was up-regulated by both ABA and NAA at E-L 35 stage. In addition, we also found that several genes encoding AUX1, TIR1, AUX/IAA, ARF, and GH3 were down-regulated by NAA at E-L 36 stage.

#### 2.1.3. Strong Transcriptional Changes of Ripening Switch Genes

**Figure 4:**
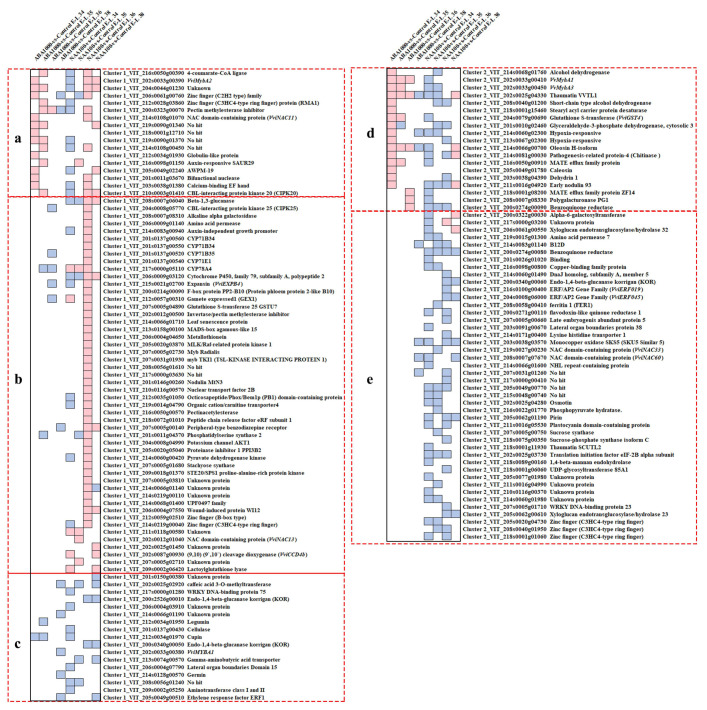
Differentially expressed switch genes between treatments and control. The significance of differentially expressed genes was judged based on the False Discovery Rate ≤ 0.01 and absolute value of log2Ratio ≥ 1. Blue and cyan boxes indicate significantly downregulated and upregulated genes in treated berries compared to the control, respectively. Genes were divided into groups (**a**–**e**) according to their different responses to the treatments.

A previous study has identified 190 grapevine berry switch genes, which trigger the onset of the ripening process [16]. All those genes were observed to express at low levels during the immature phase and show a significant increase at veraison. They were mainly involved in transcription activation, cell wall metabolism and the development process. In the present study, the expression pattern of the switch genes was investigated to detect the roles of these genes in varied ripening procession induced by ABA and NAA treatments. After removing the switch genes with low expression (RPKM < 1) among our samples, the remaining 184 switch genes were performed K-means clustering analysis, and two clusters were generated (Figure S1). The expression of 107 genes in cluster 1 kept increasing from E-L 34 to E-L 38 stage in all samples and these genes expressed at a higher level in NAA-treated berries at E-L 36 stage. In the contrast, the transcript abundance of 77 genes in cluster 2 increased at the early stage then decreased, and these genes showed lower expression level under NAA treatment. Among the switch genes of cluster 1 and 2, the 147 significant differentially expressed switch genes (DESGs) between treatments and control were shown in Figure 4, and the biological process annotation of these DESGs was listed in Table S1. According to their distinct responses to the treatments, the DESGs in the above-mentioned cluster 1 and cluster 2 were roughly divided into 3 (a–c) and 2 (d–e) groups, respectively. Interestingly, we found most genes in the group a and d were up-regulated by ABA at E-L 34 or E-L 35 stage, while down-regulated by NAA at the early stages. These genes encompassed TFs of VviMYBA2 (VIT_202s0033g00390) and zinc finger family genes (VIT_206s0061g00760 and VIT_212s0028g03860) in group a, as well as VviMYBA1 (VIT_202s0033g00410) and VviMYBA3 (VIT_202s0033g00450) in group d. Additionally, a total of 50 genes in group b were only up-regulated in NAA-treated berries particularly at E-L 36 stage. The most overrepresented biological process in this group is “Secondary Metabolic Process”, including the genes of cytochrome P450 family, glutathione S-transferase and carotenoid cleavage dioxygenase 4b (CCD4b). The genes in the group of c and e showed lower levels of expression at specific stages under NAA treatment, and they are mainly related to the transcription factor activity and cell wall metabolism. The TFs of VviWRKY75 (VIT_217s0000g01280), VviWRKY23 (VIT_207s0005g01710), VviNAC33 (VIT_219s0027g00230), VviNAC60 (VIT_208s0007g07670), three zinc finger proteins (VIT_205s0020g04730, VIT_208s0040g01950 and VIT_218s0001g01060) and two lateral organ boundaries proteins (VIT_206s0004g07790 and VIT_203s0091g00670), were found in these genes. It also included several genes encoding cellulase (VIT_201s0137g00430), endo-1,4-beta-glucanase (VIT_200s2526g00010, VIT_200s0340g00050 and VIT_200s0340g00060) and xyloglucan endotransglucosylase/hydrolase (VIT_206s0061g00550 and VIT_205s0062g00610) that are involved in fruit softening [25,26]. Moreover, among these NAA-inhibited genes, three genes are involved in carbohydrate metabolism (glycolysis and sucrose biosynthesis), including phosphopyruvate hydratase (VIT_216s0022g01770), sucrose synthase (VIT_207s0005g00750), sucrose-phosphate synthase (VIT_218s0075g00350).

### 2.2. Regulation of ABA and NAA on the Biosynthesis and Accumulation of Norisoprenoids

#### 2.2.1. Effects on Norisoprenoid Production and Related Gene Expression

**Figure 5:**
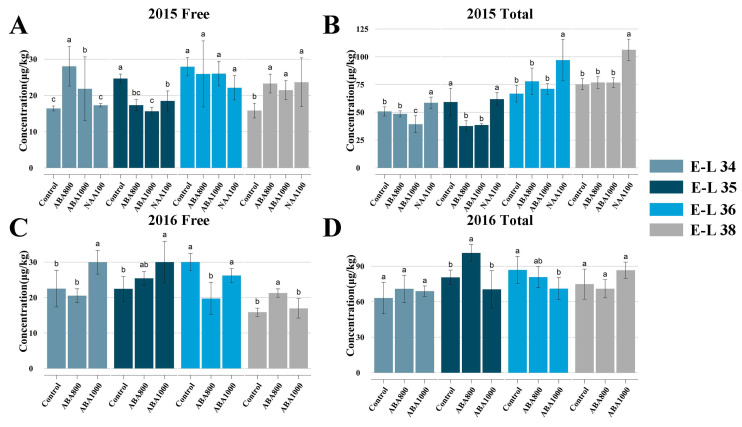
Concentrations of free, total and individual norisoprenoids. (**A**) Concentrations of free norisoprenoids in 2015. (**B**) Concentrations of total norisoprenoids in 2015. (**C**) Concentrations of free norisoprenoids in 2016. (**D**) Concentrations of total norisoprenoids in 2016. Statistical analysis involved the comparison of treatments at each E-L stage, and different letters represent significant differences (*p* = 0.05).

The concentration of free-form and total norisoprenoids varied among the berries of the treatments and the control (Figure 5). ABA1000 and ABA800 application were observed to markedly increase the concentration of free-form norisoprenoids in 2015 at E-L 34 and E-L 38 stages. Despite a similar pattern was observed in 2016, the difference between the control and ABA1000-treated berries were significant only at E-L 34 stage. Lower levels of total norisoprenoid were observed in ABA-treated berries than Control berries at E-L 34 and E-L 35 stages in 2015, higher levels were observed in ABA800-treated berries than Control berries at E-L 35 stage in 2016. No effects of ABA treatments on the total norisoprenoid concentration were observed at harvest (E-L 38). NAA100-treated berries showed a high level of both free-form and total norisoprenoids at E-L 38 in 2015. The total concentration of each norisoprenoid was presented in Table S2. When grape berries reached maturity (E-L 38 stage), a different response to the ABA and NAA treatments among the identified norisoprenoids was found. Higher concentrations of vitispirane A, vitispirane B and (E)-1-(2,3,6-trimenthylphenyl)buta-1,3-diene (TPB) were observed in NAA-treated berries at all sampling stages. Most norisoprenoid compounds such as β-damascenone and β-ionone appeared to be significantly increased by NAA treatment in harvested berries in 2015, except for geranylacetone and 6-methyl-5-hepten-2-one (MHO). ABA800 and ABA1000 had consistent negative effects on the accumulation of vitispirane A, vitispirane B and MHO in harvested berries in both seasons. Additionally, geranylacetone concentration was not influenced by ABA treatments at E-L 38 stage, and the responses of other norisoprenoids to ABA were not consistent among the two seasons. The transcriptome analysis was conducted on the ABA1000, NAA100 and control berries collected in 2015. Concerning the genes involved in norisoprenoid biosynthesis (Figure 2B), we found that ABA treatment markedly suppressed the expression of VviLECY, VviCCD4a and VviABA-Hase at the early stage, but elevated the expression of VviCCD4b and key ABA biosynthesis-related genes including VviNCED1, VviNECD2, and VviNCED3. Expectedly, NAA treatment up-regulated the norisoprenoid biosynthesis-related genes such as VviPSY1, VviPSY2, VviPSY3, VviLECY, VviLBCY, VviZEP, VviCCD4a, and VviCCD4b, which corresponded to the increase in total concentration of norisoprenoids. Meanwhile, this treatment also suppressed the expression of VviPSY (VIT_203s0038g00450) at E-L 36 stage, three VviNCEDs at E-L 34 stage and two VviABA-Hases. The transcription of other norisoprenoid genes such as PDS, ZISO, CRTISO, LUT5/BCH, LUT1, AAO, and CCD1 were insensitive to both ABA and NAA treatments.

#### 2.2.2. Acquisition of the Candidate Genes Regulating Norisoprenoid Accumulation

**Figure 6:**
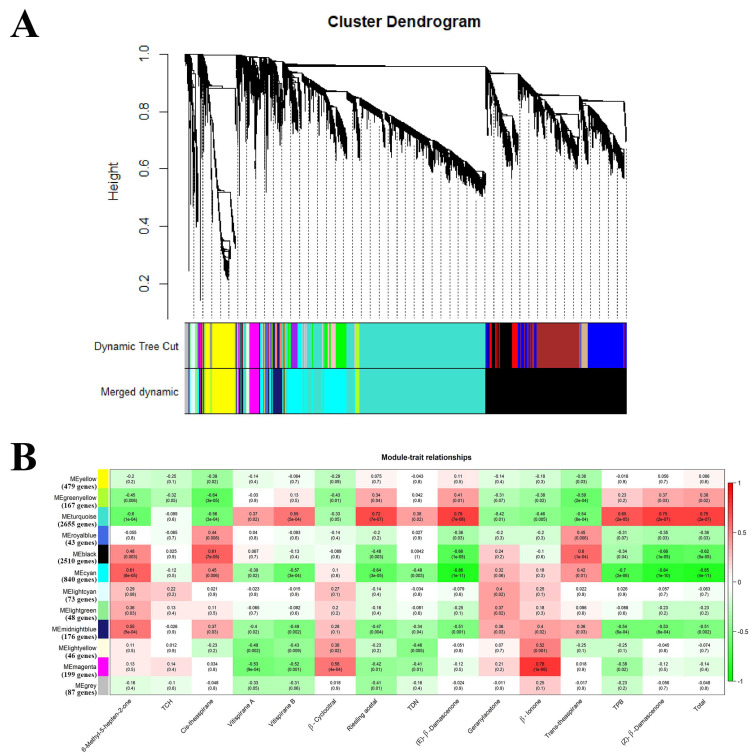
Weighted gene co-expression network analysis (WGCNA) of differentially expressed genes (DEGs) induced by ABA100 or NAA100 and the hierarchical cluster analysis of associated genes related to the accumulation of norisoprenoid. (**A**) Hierarchical cluster tree showing 12 merged modules of co-expressed genes. (**B**) Module-trait correlations and corresponding *p*-values. The left panel shows 12 modules and the right panel is a color scale for module trait correlation from −1 to 1.

To identify the ABA and NAA-responsive transcription factors regulating norisoprenoid biosynthesis, an effective system biology method called weighted gene co-expression network analysis (WGCNA), was performed to find the modules of highly correlated genes and relate these modules to traits. For each module, eigengene is defined as the first principal component of the expression matrix of the module and considered to be representative of the module gene expression profiles. It is then used to correlate with traits and look for the significant association. Based on the genes differentially expressed between the treatment and control at least one phenological stage, twelve modules were established (Figure 6A). The top intramodular hub genes of each module were defined by using the intramodular connectivity measure. Among the 12 modules, the modules of “turquoise”, “cyan”, “black”, and “magenta” were found to be associated with norisoprenoid accumulation. The module turquoise showed a strong correlation with the concentration of riesling acetal (r = 0.72, *p* = 7 × 10^−7^), (E)-β-damascenone (r = 0.76, *p* = 7 × 10^−8^), (Z)-β-damascenone (r = 0.75, *p* = 2 × 10^−7^) and total norisoprenoids (r = 0.75, *p* = 2 × 10^−7^), and moderately correlated with 6-methyl-5-hepten-2-one (r = −0.60, *p* = 1 × 10^−4^), cis-theaspirane (r = −0.56, *p* = 3 × 10^−4^), vitispirane B (r = 0.55, *p* = 5 × 10^−4^), trans-theaspirane (r = −0.54, *p* = 8 × 10^−4^) and TPB(r = 0.65, *p* = 2 × 10^−5^) contents (Figure 6B). The module–trait relationship analysis also identified module cyan as most highly related to (E)-β-damascenone (r = −0.86, *p* = 1 × 10^−11^), TPB (r = −0.70, *p* = 2 × 10^−6^), (Z)-β-damascenone (r = −0.84, *p* = 1 × 10^−10^) and total norisoprenoids (r = −0.85, *p* = 4 × 10^−11^). The module black exhibited a moderate correlation with cis-theaspirane (r = 0.61, *p* = 7 × 10^−5^), (E)-β-damascenone (r = −0.66, *p* = 1 × 10^−5^), trans-theaspirane (r = 0.60, *p* = 1 × 10^−4^), (Z)-β-damascenone (r = −0.66, *p* = 1 × 10^−5^) and total norisoprenoids (r = −0.62, *p* = 5 × 10^−5^). Moreover, the moderate correlation of β-cyclocitral (r = 0.56, *p* = 4 × 10^−4^) and high correlation of β-ionone (r = 0.78, *p* = 1 × 10^−8^) were observed in magenta module. Interestingly, the VviCCD4a and VviCCD4b which have been reported to tightly associate with norisoprenoid accumulation [7], were presented in this module turquoise and cyan, respectively. Furthermore, it was found that VviCCD4a co-expressed with the upstream genes of norisoprenoid biosynthesis including VviPSY2, VviPSY3 and two VviZEPs in module turquoise, while VviCCD4b co-expressed with VviNCED1, VviNCED2, and VviNCED3 in module cyan. The VviPSY1, VviZDS (VIT_214s0030g01740) and VviLECY were clustered in the module black. However, in module magenta, there were no known genes related to norisoprenoid biosynthesis. Therefore, to investigate the regulation of norisoprenoid biosynthesis, the top 30 hub genes in modules of turquoise, cyan, and black were further analyzed. Among those hub genes (Table S3), only one transcription factor of VviGATA26 (VIT_200s2393g00010) was found and it was in module turquoise. This TF could be potentially involved in the regulation of norisoprenoid biosynthesis.

To characterize the VviGATA26 function, DNA affinity purification sequencing (DAP-seq) was performed to understand the binding sites of VviGATA26 in the grape genome. Totally 6785 binding locations (peaks) were found, among which 1872 binding sites were localized in the promoter region of 1817 target genes. The target genes also included those genes encoding VviPSY1 (VIT_204s0079g00680), VviZDS (VIT_214s0030g01740), and VviABA-Hase (VIT_204s0079g00680), which were participated in the norisoprenoid biosynthesis (Figure 2B). However, we noticed that VviPSY1 and VviZDS were included in black module, which negatively correlated with the concentrations of (E)-β-damascenone, (Z)-β-damascenone, and total norisoprenoids. The VviABA-Hase was in “midnightblue” module, but this module did not exhibit a high correlation with norisoprenoid. Additionally, KEGG enrichment analysis revealed that the target genes predicted for VviGATA26 were significantly enriched in the pathway of “plant hormone signal transduction”. In this case, VviGATA26 could also be involved in the signaling regulation of hormones including ABA, auxin, cytokinine, gibberellin, salicylic acid, brassinosteroid, jasmonic acid, and ethylene (Table S4).

#### 2.2.3. An Integrated Gene Co-Expressed and Regulatory Network Regulating Norisoprenoid Biosynthesis

Our results indicate that ABA and NAA treatments can significantly influence the expression of norisoprenoid-related genes, previously identified switch genes, and the genes involved in ABA and auxin biosynthesis and signaling nearly at the same time. Therefore, we hypothesized that there is a crosstalk between norisoprenoid-related genes and the other genes. The potential link between these genes is supported by the study demonstrating norisoprenoid-related gene VviCCD4b (VIT_202s0087g00930) is a berry switch gene [16]. Furthermore, the ABA and norisoprenoid share the common substrate of carotenoids, and hence are under the regulation of the same upstream genes. Therefore, we integrated the results of WGCNA and DAP-seq analysis to build a gene co-expressed and regulatory network, the objective of which was to improve our understanding of the regulation of norisoprenoid accumulation.

**Figure 7:**
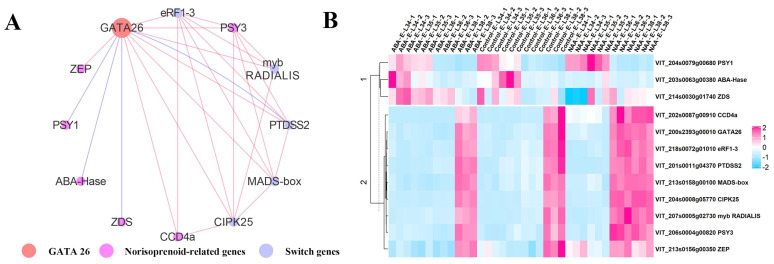
Gene co-expressed and regulatory network. (**A**) Gene co-expressed and regulatory network related to norisoprenoid biosynthesis. The size of the nodes corresponds to the nodes ‘betweenness centrality’, which measures how often a node in the network occurs on all shortest paths between two nodes and help identify the gene that plays a ‘bridge spanning’ role in the network. Red and blue edges indicate that the interaction between two genes is identified by WGCNA and DNA affinity purification sequencing (DAP)-seq, respectively. (**B**) Hierarchical cluster analysis of genes in the left graph. The colors of the boxes represent the intensity of the normalized gene.

In the norisoprenoid-related WGCNA modules of turquoise, cyan, and black, we observed some switch genes and hormone-related genes exhibited similar expression patterns with the genes involved in norisoprenoid biosynthesis (Table S5). A high edge weight threshold of 0.4 was chosen to select the candidate genes to construct the network within these modules. After removing the edges according to the threshold, the gene interactions observed in the network were all come from the module of turquoise. The norisoprenoid-related genes of VviPSY1, VviZDS (VIT_214s0030g01740), and VviABA-Hase (VIT_204s0079g00680), which were the target genes of VviGATA26 identified by DAP-seq, were also included in the network. It was found that VviCCD4a co-expressed with VviGATA26, and two switch genes encoding eukaryotic peptide chain release factor subunit 1-3 (eRF1-3, VIT_218s0072g01010) and CBL-interacting protein kinase 25 (CIPK25, VIT_204s0008g05770) (Figure 7A). In addition to these three genes, the VviPSY3 interacted with the other three switch genes encoding myb RADIALIS (VIT_207s0005g02730), phosphatidylserine synthase 2 (PTDSS2, VIT_201s0011g04370), and MAD-box (VIT_213s0158g00100). Additionally, the heatmap clearly exhibited that VviGATA26 expression was up-regulated at the E-L 36 stage of NAA-treated grape berries. A relationship between VviZEP and VviGATA26 was also observed in the network. The expression pattern of these genes in all samples suggested VviGATA26 could negatively regulate the expression of the DAP-seq identified genes of VviPSY1, VviZDS, and VviABA-Hase in cluster 1, while positive correlations were found between pairs of genes in cluster 2 (Figure 7B).

#### 2.2.4. Potential Contribution of Differentially Alternative Splicing of VviDXS and VviCRTISO

**Figure 8:**
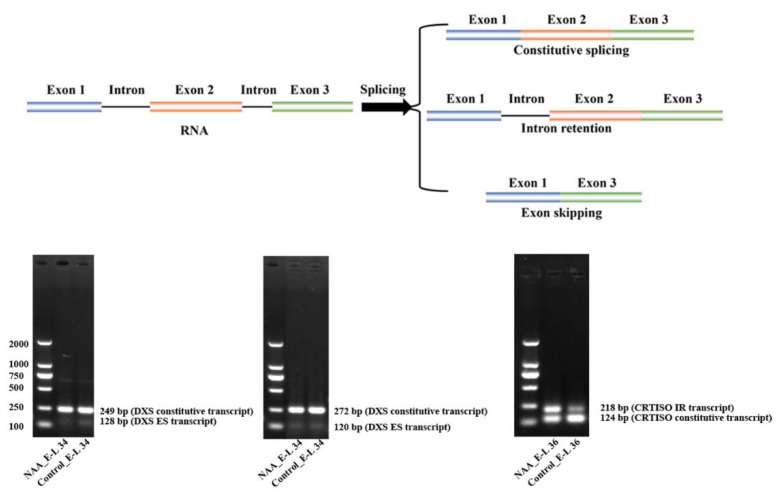
Qualitative RT-PCR analysis of expression of *VviDXS* and *VviCRTISO* splice variants in grape berries at specific stage under NAA100 and ABA1000 treatments. The forward and reverse primers were designed from the upstream and downstream exons of the skipped exon or retention intron, respectively.

The effects of the ABA and NAA treatments on alternative splicing (AS) events of those genes were also investigated. As a posttranscriptional regulation of genes, AS has been shown to be affected by salt stress and high temperature in grape berry [9,11]. Splice variants are mainly generated by intron retention (IR), exon skipping (ES), mutually exclusive exon (MXE), alternative 3′ splice site (A3SS) and alternative 5′ splice site (A5SS). The differential alternative splicing between two RNA-Seq samples was detected by rMATs [27]. In the present study, besides the norisoprenoid-associated genes mentioned above, the upstream genes involved in the plastidial 2-methyl-D-erythritol-4-phosphate phosphate (MEP) and cytoplasmic mevalonic acid (MVA) pathways were also considered [28]. Among these genes, there were 32 differentially splicing ES or IR events were found (Tables S6 and S7). However, only the occurrence of alternative splicing at the genes encoding 1-deoxy-D-xylulose-5-phosphate synthase (DXS; VIT_204s0008g04970) and prolycopene isomerase (CRTISO; VIT_208s0032g00800) was further validated by reverse transcription PCR (Figure 8). The rMATS paired model identified that the two transcripts of the VviDXS with different ES were up-regulated by NAA at the stage of E-L 34 (Table S6), and a transcript of the VviCRTISO with IR was also expressed at relatively high levels in NAA-treated berries compared to the control at E-L 36 stage (Table S7).

## 3. Discussion

### 3.1. Response of ABA and IAA Biosynthesis and Signaling

This study deals with the responses of endogenous ABA and auxin biosynthesis and signaling, and previously identified switch genes to exogenous ABA and NAA treatments (Figure 2, Figure 3 and Figure 4). These data provide a framework to understand numerous aspects of ABA and NAA-regulated ripening. We found that the level of endogenous ABA significantly increased after ABA treatments in 2015 (Figure 2A), which is consistent with the previous study [20]. The elevated ABA could result from both induced endogenous ABA biosynthesis and the absorb from exogenous spraying ABA. However, the uptake of exogenous ABA into the grape was predicted to be an inefficient process because of the berry waxy cuticle [20,29]. Given the increased ABA mainly came from ABA biosynthesis, a small amount of external ABA entering the berry may be enough to drive the flux of carotenoid into endogenous ABA production. This speculation is supported based on the upregulation of VviNECDs in ABA-treated berries **(**Figure 2B), as well as the higher correlation between ABA concentration and VviNCED1 expression (r = 0.79, *p* = 0.011) or VviNCED2 expression (r = 0.70, *p* = 0.036). However, auxin analogues NAA had both positive and negative effects on the expression of auxin biosynthetic genes (Figure 2C), and this explained why there was no significant difference in IAA concentration between NAA treatment and the control. From the aspect of interactive crosstalk, the ABA treatment inhibited the endogenous auxin production by suppressing the expression of VviTAR1 and VviYUC10, and NAA application suppressed endogenous ABA biosynthesis by synchronously down-regulating three VviNCEDs at E-L 34 stage (Figure 2B). The lower expression of ABA biosynthetic genes in NAA-treated berries has been also observed in a previous study [30].

The ABA and NAA treatments also caused changes in the transcription rate of ABA and auxin signaling genes. As a whole, ABA spraying activated the ABA signaling transduction and suppressed the auxin signaling transduction, whereas the effects of NAA treatment on the two signaling pathways appeared to be complex, owing to the up-regulation or down-regulation of various genes (Figure 3). The complicated regulatory network between ABA and auxin signaling pathways has been already suggested by Nemhauser et al. [31] and Fiorenza et al. [30]. As expected, some ripening switch genes previously identified in grape berry were found to have the opposite expression behavior in response to ABA and NAA treatments, such as the DESGs in the group of a and d (Figure 4b). Indeed, VviMYBA2, VviMYBA1, VviMYBA3, and zinc finger family genes in the groups of a and d have been demonstrated to involve in berry development and ripening [32,33]. It was considered that these TFs were up-regulated by ABA while down-regulated by NAA treatment at E-L 34 or E-L 35 stage, in turn, which monitored grape ripening. Moreover, the delayed ripening of NAA-treated berries could also attribute to the switch genes in c and e groups that were significantly down-regulated by NAA while almost unchanged in ABA-treated berries. In these two groups, the potential ripening regulators of VviWRKY75, VviNAC33, VviNAC60, and two lateral organ boundaries proteins were found to be associated with ripening regulation (Table S1). In addition to TFs, we found that the expression of genes concerning fruit softening and carbohydrate metabolism in group c and e was inhibited by NAA spraying.

### 3.2. Response of Norisoprenoid Biosynthesis

Our study is the first to reveal the effect of exogenous ABA on both free and total norisoprenoids in grape berries at both transcriptional and post-transcriptional levels. As is known, the biosynthetic pathways of ABA and norisoprenoid share the same part of the substrate of carotenoids. The present study indicated that though ABA biosynthesis was enhanced by ABA treatment, there is no visible decrease of total norisoprenoids in ABA-treated harvested berries. It was found the total norisoprenoid levels are low in both ABA1000 and ABA800-treated berries only at E-L 34 stage (Figure 5A,B) when the concentration of ABA largely increased (Figure 2A). The previous researchers have confirmed the increase of carotenoids after ABA treatment in many fruits such as in grapes [34] and tomato [35] and seeds of bean, tobacco, beet, and corn [36]. Hence, we speculated that ABA treatment elevated the concentration of carotenoids or ABA precursors during berry ripening, thus supporting the biosynthesis of both norisoprenoids and ABA. Alternatively, since vitispirane A, vitispirane B, and MHO exhibited lower levels in ABA-treated berries in consecutive two seasons (Table S2), ABA treatments or the increased endogenous ABA may negatively regulate the accumulation of these compounds by down-regulating VviCCD4a (Figure 2B). The pre-veraison NAA treatment was found to increase total norisoprenoids at E-L 35 and E-L 36 stages in grape berries [21] and β-damascenone level in wine [3]. Different from ABA treatment, NAA effect on norisoprenoid biosynthesis, by comparison to ABA biosynthesis, appeared to be more apparent. NAA up-regulated a series of norisoprenoid-associated genes including VviPSY1, VviPSY2, VviPSY3, VviLECY, VviLBCY, VviZEPs, VviCCD4a, and VviCCD4b expression at a certain stage (Figure 2B), ultimately elevating the level of most norisoprenoid components at harvest, except for geranylacetone and 6-methyl-5-hepten-2-one. In tomato fruit, silencing of PSY1 can significantly reduce carotenoid accumulation, while the silencing of PSY2 or PSY3 was less efficient in controlling carotenoid biosynthesis [37], suggesting the three PSY genes played different roles in the carotenoid biosynthetic pathway. Similarly, since carotenoids are synthesized mostly from fruit formation until veraison in grape berry [1], the higher expression of VviPSY1 at E-L 34 and E-L 35 stages (Figure 7B) indicated its important role in carotenoid metabolism. In contrast, the elevated expression levels of VviPSY2 and VviPSY3 were observed at E-L 36 and E-L 38 stages, which could be responsible for the carotenoid biosynthesis after veraison. It may explain the different responses of VviPSYs to NAA treatments (Figure 2B). WGCNA further expounded that VviCCD4a expression had a high correlation with (E)-β-damascenone, a norisoprenoid component with the highest concentration, and (Z)-β-damascenone, riesling acetal, TPB, as well as total norisoprenoid concentration (Figure 6), while VviCCD4b was correlated only with the concentration of 6-methyl-5-hepten-2-one. This result is different from our previous study in which we found that total norisoprenoids and β-damascenone were positively correlated with the expression of VviCCD4b, rather than VviCCD4a during Cabernet sauvignon grape berry development [12]. This difference is inferred to mainly relate to the ripening process retarding and transcriptional alteration induced by NAA. In the present study, the expression of VviCCD4a was remarkedly up-regulated by NAA treatment at E-L 34, E-L 35, and E-L 36 stages, but was down-regulated by ABA spraying at E-L 35 and E-L 36 stages (Figure 2B). Moreover, VviCCD4a co-expressed with the upstream VviPSYs and VviZEPs of norisoprenoid biosynthesis pathway (Table S5). Both our present data and previous finding [12] indicated that VviCCD4a in the non-treated grape berries is expressed at a very low level before E-L 36 stage, and this gene expression is sharply increased when berries approach technical maturity (Figure S2). This study also observed that the expression level of VviCCD4a was much higher than VviCCD4b in NAA-treated berries at E-L 34, E-L 36, and E-L 38 stages. Taken together, these results indicate that VviCCD4a should be a key enzyme affecting the norisoprenoid accumulation in response to NAA treatment.

### 3.3. Potential Regulation Relating to NAA-Induced Norisoprenoid Accumulation

GATA transcription factors are a family of zinc finger proteins that bind the consensus DNA sequence (T/A) GATA (A/G) [38]. They are widely present in plants and involved in light response regulation, chlorophyll synthesis, and carbon/nitrogen metabolism. Interestingly, Evidence has demonstrated that light is the most important environmental factors affecting plant carotenoid metabolism; light significantly promotes the expression of carotenoid biosynthetic genes, particularly PYS and the activity of the related enzymes [39]. In the present study, integrated WGCNA and DAP-seq analysis indicated that VviGATA26 played a critical role in the regulatory network relating to NAA-induced norisoprenoid biosynthesis based on the following data. Firstly, VviGATA26 was indicated to be able to bind with the promoter sequence of VviPSY1 (VIT_204s0079g00680) and VviZDS (VIT_214s0030g01740), and VviGATA26 exhibited an opposite expression pattern with the two target genes at the transcriptional level (Figure 7B). Both VviPSY and VviZDS are crucial enzymes in the biosynthesis of norisoprenoid precursor of carotenoid [40]. As the rate-limiting step of carotenogenesis, VviPSY1 has garnered much attention and numerous strategies targeting PSY1 have been performed to increase carotenoid concentration in tomato [41]. Over-expression of AtZDS in tomato resulted in the increased carotenoid all trans-lycopene, and reduced carotenoid content was found in ZDS repressed fruit [42]. Secondly, the expression of these two target genes, especially VviPSY1, was reversely paralleled with the accumulation of (E)-β-damascenone, (Z)-β-damascenone, and total norisoprenoids (Table S8). Thirdly, the higher expression of VviGATA26 expression was showed in NAA-treated berries at E-L 36 stage compared to the control (Figure 7B). Combining these three points, we hypothesized that VviGATA26 could positively respond to NAA treatment and be triggered to down-regulate the two upstream genes and resulted in the increased accumulation of norisoprenoids. In addition, VviGATA26 expression was positively correlated with VviCCD4a, VviPSY2, VviPSY3 and VviZEPs (Figure 7B), but VviGATA26 cannot bind to the promoter regions of these genes according to DAP-seq. A possible explanation is that VviGATA26 indirectly affects the expression of these genes via regulatory cascades. Two TFs VviMYB RADIALIS (VIT_207s0005g02730) and VviMADS-box (VIT_213s0158g00100) were also precited to involve in norisoprenoid accumulation based on their co-expression patterns with VviPSY3. Both MYB and MADS-box transcription factor families have been demonstrated to associate with ripening process modulation [43,44]. Our previous study has elucidated that MADS4 (VIT_201s0010g03900) can participate in the regulation of norisoprenoid accumulation by negatively regulating VviCCD4b [12]. In citrus, a MADS transcription factor, CsMADS6 was reported to be able to bind to the promoter of PSY and up-regulated its expression [45]. However, the present data is far from supporting our hypothesis regarding the regulatory function of the VviMYB RADIALIS and VviMADS-box in norisoprenoid biosynthesis. In future work, the genome-editing techniques or transgenic grapevines will be used to verify this point.

AS, as an important posttranscriptional regulation of genes, has been reported to happen in 8668 genes of v2 predicted genes (29150) in grape berry [9]. As observed in grape and Arabidopsis [11,46], AS contribute significantly to the transcriptional complexity and should be taken into consideration when performing genome wider transcriptomic studies. The expression of the isoforms of VviDXS and VviCRTISO was significantly altered in the comparison of NAA and control, which may then affect the protein properties and finally contribute to higher norisoprenoid. It would be interesting and useful to further investigate if the AS produced transcript will be biologically functional in the future, although how to distinguish aberrant and functional splicing remains an unresolved question [47]. The integration of transcriptomics and metabolomics with proteomics could serve as a starting point to identify functional AS events in grape and other plants.

## 4. Materials and Methods

### 4.1. ABA and NAA Treatments and Sampling

Clusters of *Vitis vinifera* L. cv. Cabernet Sauvignon grapevines cultivated in Shanxi Academy of Agricultural Sciences Pomology Institute, Shanxi, China, in 2015 and 2016, were used for the study. The 1000 mg/L ABA, 800 mg/L ABA, and 100 mg/L NAA solutions containing 0.05% Tween 20 were applied at seven weeks after flowering (berry still hard and green, E-L 33 stage), with 0.05% Tween solution as the control. The spray of these solutions was performed at sunset to avoid the rapid evaporation of the solutions. After the first spray solution, the second application was conducted ten hours later on the same day. A randomized block design was considered for this study, and each treatment or control was replicated in three plots of 50 vines. Each replicate included 500 berries collected from 50 vines. Berry sampling was performed at four E-L stages (E-L 34, E-L 35, E-L 36, and E-L 38) according to the modified E-L system [48]. The leaves at E-L 34 stage were collected for DNA extraction. All samples were kept in dry ice and immediately transferred to the laboratory. Before the extraction, samples were washed with distilled water to remove the unabsorbed ABA and NAA residues on the berry surface. Approximately 30 berries were used for the total soluble solids (TSS) and titratable acidity analysis and the others were frozen using liquid nitrogen and stored at −80 °C for further analysis.

### 4.2. Measurements of Total Soluble Solids and Titratable Acid

The total soluble solids and titratable acid (expressed as g tartaric acid equivalents per liter of juice) were measured using a digital handheld pocket Brix refractometer (PAL-2, ATAGO, Tokyo, Japan) and adjusting the pH of the grape juice to 8.2 using NaOH, respectively.

### 4.3. RNA Extraction and Sequencing

The berry samples used for RNA sequencing including the berries of the control, ABA1000-treated and NAA-treated at E-L 34, E-L 35, E-L 36, and E-L 38 stages in 2015. Total RNA was isolated using a plant RNA isolation kit and following the manufacture’s protocol (Sigma RT-250, St. Louis, MO, USA), and quality and quantitation of RNA were assessed by a Qubit 2.0 fluorometer RNA Assay Kit (Invitrogen Inc., CA, USA) and Agilent 2100 Bioanalyzer (Agilent, Santa Clara, CA, USA). Totally 36 RNA-seq libraries were constructed (each sample has three biological replicates) using Illumina Hiseq X Ten (Illumina Inc., San Diego, CA, USA) to yield 150-bp pair-end reads.

### 4.4. Extraction and Determination of ABA and IAA

For each biological replicate, 10 g deseeded grape berries were ground into powder under liquid nitrogen. The extraction and quantification of ABA and IAA were performed according to the published method [49]. Briefly, 100 mg powder was weighed into a 1.5 mL centrifuge tube, then the powder with 750 µL water added was placed in an ultrasonic cleaner for 30 min. After centrifugation at 15,000 rpm for 10 min, the supernatant was transferred to a new tube and 750 µL MeOH-ACN (1:1, *v*/*v*) was added. Then sonication and centrifugation were performed. The 400 µL mixed supernatant was dried using a vacuum concentrator and re-dissolved in aliquots of 80 µL MeOH-H2O (1:1, *v*/*v*), filtered through a 0.1 µm membrane and transferred to vials for LC-MS analysis. Quantification was performed using a UPLC-HRMS system (UPLC, ACQUITY UPLC H-Class Bio, Waters; MS, Q-Exactive, Thermo Scientific, Bremen, Germany) coupled with heated electrospray ionization (HESI) source. UPLC separation was performed on a BEH C18 column (2.1 × 100 mm, 1.7 µm) at a flow rate of 0.3 mL min^−1^. The mobile phases were composed of 0.1% FA in water (phase A) and 0.1% FA in ACN (phase B). The following gradient program was applied: 95% A at 0 min to 55% A at 7 min, 5% A at 10 min and held for 4 min, then returned to the initial condition. ABA and IAA were used as external standards to quantify ABA and IAA levels in grape berries.

### 4.5. Analysis of Norisoprenoids in Berries Using SPME-GC-MS

About 50 g berries with the seed removed were blended with 1 g PVPP and ground into powder in liquid nitrogen. The extraction of free and total norisoprenoid was conducted following published research with some modification [50]. For free norisoprenoid, we weighted 1 g berry powder and put it into a 20 mL autosampler vial with 5 mL of citrate buffer (0.2 M, pH 3.2, saturated with NaCl) and 10 µL internal standard (1.008 mg/L 4-Methyl-2-pentanol) added. Then the vials were tightly capped and equilibrated at 50 °C in a thermostatic bath for 15 min. To extract total norisoprenoid, 1 g berry powder was put into a 20 mL autosampler vial with 5 mL of citrate buffer (0.2 M, pH 2.5, saturated with NaCl) and 10 µL internal standard (1.008 mg/L 4-Methyl-2-pentanol) added. Then the vials were tightly capped and equilibrated at 99 °C in a thermostatic bath for 1 h.

The volatile compounds were extracted by headspace-solid phase microextraction (HS-SPME) using 2 cm DVB/CAR/PDMS 50/30 μm SPME fiber (Supelco, Bellefonte, PA, USA) at 40 °C for 30 min with stirring. An Agilent 6890 gas chromatography coupled with an Agilent 5975C mass spectrometer was used to analyze the volatile compounds in the samples according to the method described by Wang et al. [51]. The compound separation was achieved with an HP-INNOWAX capillary column (60 m × 0.25 mm × 0.25 μm, J & W Scientific, Folsom, CA, USA). Volatile compounds were identified by comparing mass spectrums and retention time with the available external standards. The compounds with reference standards were identified by comparing their retention indices and mass spectrums with the NIST11 database. Quantitation followed our published method [52]. The volatile compounds with available standards were quantified based on their reference standards, whereas the volatiles without available standards were quantified using standards that had the same functional groups and/or similar numbers of carbon atoms.

### 4.6. RNA Isolation, Cloning, and Expression of VviGATA26

Total RNA was extracted from grape berry using the plant DNA isolation kit (Sigma-Aldrich, St. Louis, MO, USA) according to the manufacturer’s instructions. The quality and concentration of RNA were detected by agarose gel electrophoresis using a NanoDrop 2000 spectrophotometer (Thermo Fisher Scientific, MA, USA). First-strand cDNA was synthesized from 1 mg total RNA in a 20 µL reverse transcription reaction mixture following the protocol of HiScript R II Q RT SuperMix for qPCR C gDNA wiper (Vazyme, Nanjing, China). PCR cloning of full-length *VviGATA26* was performed in a total volume of 25 μL containing 1 μL of cDNA template, 2 μL RT-PCR primers, 12.5 μL 2 × Taq PCR MasterMix (KT201) (Tiangen Biotech, Beijing, China) and 9.5 mL ddH2O. A pair of primers (forward: ATGGTACCTTCAAGGAAGAG, reverse: TCAGGGACGCAAAAGATGTG) was designed using primer 5.0 based on the nucleotide sequence of *VviGATA26* (XM_003635597.2) from the NCBI. The PCR product was gel purified and then ligated to the pMD18-T vector (Takara, Beijing, China) for DNA sequencing. The coding sequencing of *VviGATA26* was cloned into a pFN19K HaloTag T7 SP6 Flexi expression vector. TNT SP6 Coupled Wheat Germ Extract System (Promega, Madison, WI, USA) was used for Halo–PeWRKY1 fusion protein expression following the manufacturer’s specifications for expression in a 50 μL reaction with a 2 h incubation at 37 °C. Expressed proteins were directly captured using Magne Halo Tag Beads (Promega, Madison, WI, USA).

### 4.7. DAP Affinity Purification Sequencing

Genomic DNA was extracted from leave tissues of Cabernet Sauvignon grapevines using a one-step plant DNA extraction reagent (Bio Teke, Beijing, China). The DNA was dissolved in 50 μL of Tris-EDTA buffer. DNA-seq binding assays were performed as described by a previous study [53]. Sequencing was performed on an Illumina NavoSeq. Reads were mapped to the grape reference genome sequence using BOWTIE2 and annotated in comparison with the V2.1 version (http://genomes.cribi.unipd.it/grape/). Peak calling was conducted using Macs2. Association of DAP-seq peaks located upstream or downstream of the transcription start site within 2 kb were analyzed using Homer, according to the General Feature Format (GFF) files. Gene function annotation was blasted from NT, NR, Swissprot, and Pfam databases. FASTA sequences were obtained using BEDTools for motif analysis and motif discovery was performed using MEME-Chip suite (http://meme-suite.org/tools/meme-chip).

### 4.8. RT-PCR Analysis of Alternative Splicing

The reverse transcription polymerase chain reaction (RT-PCR) splicing analysis of *VviDXS* and *VviCRTISO* was performed in a 10 μL reaction volume. Each reaction contains 1 μL of the cDNA template, 0.5 μL forward primer, 0.5 μL reverse primer, 3 μL ddH2O, and 5 μL of 2 × Premix Ex-Taq polymerase (Takara). The cycling conditions were 98 °C for 30 s, followed by 40 cycles of 95 °C for 10 s, 60 °C for 30 s, and a 60 s extension at 72 °C, with a final 10 min extension at 72 °C. The PCR amplicons were analyzed using 2% agarose gel electrophoresis. The primers were designed from the upstream and downstream exons of the skipped exon or retention intron (Table S9).

### 4.9. Data Analysis

Data were expressed as the mean ± standard deviation of triplicate tests. One-way analysis of variance (ANOVA) was performed to measure the difference among the means under Duncan’s multiple range test (DMRT) at a significant level of 0.05 using the R package “agricolae”. The average number of clean reads generated by RNA sequencing was 68.04 million. Clean reads were then mapped to the grape reference genome (http://genomes.cribi.unipd.it/grape/) using TopHat. The read mapping rate all exceeded 70% for the respective RNA-seq libraries (Table S10), indicating that the sequencing quality was sufficient for further data mining. All of these reads were assembled into 25,280 genes. The normalized expression of the gene was calculated as a Reads Per Kilobases Per Million Reads (RPKM) value. The transcriptomic data are available in NCBI Gene Expression Omnibus repository (http://www.ncbi.nlm.nih.gov/geo/) under accession number GSE150343. We used the R package “DESeq2” to analyze the differentially expressed genes (DEGs), and the significance was judged based on the False Discovery Rate ≤ 0.01 and the absolute value of log2Ratio ≥ 1. K-means analysis was performed using the R package “factoextra” and “stats”, and WGCNA was conducted by R package “WGCNA”. Hierarchical clustering analysis of metabolites was performed using the expander R package “ComplexHeatmap.” All the data were analyzed with the open-source R statistical computing environment (3.6.2) in the present study.

## 5. Conclusions

In the present study, we characterized the roles of endogenous ABA and auxin biosynthesis and signaling, along with the previously identified switch genes in the ABA and NAA-induced ripening changes. The responses of free and total norisoprenoids in Cabernet Sauvignon grape berries to ABA and synthetic auxin were revealed and interpreted in both transcriptional and posttranscriptional levels. GATA26 (VIT_200s2393g00010), myb RADIALIS (VIT_207s0005g02730), and MAD-box (VIT_213s0158g00100) were identified as potential regulators of norisoprenoid accumulation by WGCNA and DAP-seq. VviGATA26 was inferred to target to down-regulate the expression of *VviPSY1* and *VviZDS*, and positively regulate the expression of *VviCCD4a*, *VviPSY2*, *VviPSY3,* and two *VviZEPs*, to benefit the norisoprenoid accumulation. The future study will focus on the molecular mechanism of GATA26 regulating the metabolism from carotenoids to norisoprenoids and the inducing function of auxin on this mechanism. From the perspectives of viticulturists and winemakers, the present findings also can give them some suggestions to improve the concentration of norisoprenoids in Cabernet Sauvignon grape berries.

## Supplementary Materials

Supplementary Materials can be found at https://www.mdpi.com/1422-0067/22/3/1420/s1, Figure S1. K-means analysis clustering of switch genes; Figure S2. Expression of *VviCCD4a* and *VviCCD4b* during berry development and ripening; Table S1. Functional annotation of differentially expressed switch genes; Table S2. Total concentrations (µg/kg) of individual norisoprenoid in 2015 and 2016; Table S3. Hub genes identified in the modules related to norisoprenoid; Table S4. Potential target genes of transcription factor VviGATA26 in plant signal transduction; Table S5. Genes in the WGCNA identified modules of ‘turquoise’, ‘cyan’ and ‘black’; Table S6. Differentially splicing ES events between treatments and control; Table S7. Differentially splicing IR events between treatments and control; Table S8. Pearson correlation coefficient r. Values in bold letters show significant correlations (t-test, α = 0.05); Table S9. The primers of RT-PCR splicing analysis; Table S10. Summary of RNA-seq mapping statistics.

## Abbreviations

ABA	Abscisic acid
AS	Alternative splicing
AAO	Abscisic-aldehyde oxidase
ABF	ABA-responsive element binding factor
AUX1	Auxin influx carrier
AUX/IAA	Auxin/indole-3-acetic acid protein
ARF	Auxin response factor
A3SS	Alternative 3′ splice site
A5SS	Alternative 5′ splice site
CCD	Carotenoid cleavage dioxygenase
CRTISO	Prolycopene isomerase
DAP-seq	DNA affinity purification sequencing
DEG	Differentially expressed gene
DXS	1-Deoxy-D-xylulose-5-phosphate synthase
DESG	Differentially expressed switch gene
ES	Exon skipping
GH3	Indole-3-acetic acid-amido synthetase
IR	Intron retention
LBCY	Lycopene beta-cyclase
LECY	Lycopene epsilon-cyclase
LUT1	Carotene epsilon-monooxygenase
LUT5	beta-ring Hydroxylase
LBD	Lateral organ boundaries
MHO	6-Methyl-5-hepten-2-one
MEP	2-Methyl-D-erythritol-4-phosphate phosphate
MVA	Mevalonic acid
MXE	Mutually exclusive exon
NAA	1-Naphthaleneacetic acid
NCED	9-*cis*-Epoxycarotenoid dioxygenase
NSY	Neoxanthin synthase
RPKM	Reads per kilobases per million reads
SAUR	Small auxin-up RNA
TF	Transcription factor
TSS	Total soluble solids
TA	Titratable acidity
TAR	Tryptophan aminotransferase related
TIR1	Transport inhibitor response 1
TAA1	Tryptophan aminotransferase arabidopsis1
PSY	Phytoene synthase
PP2C	Protein serine/threonine phosphatase 2C
PDS	15-*cis*-Phytoene desaturase
WGCNA	Weighted gene co-expression network analysis
XDH	Xanthoxin dehydrogenase
YUC	YUC flavin monooxygenase.
ZDS	Zeta-carotene desaturase
ZEP	Zeaxanthin epoxidase
ZISO	Zeta-carotene isomerase
