# Supplementary figures and images for: Combined Metabolite and Transcriptome Profiling Reveals the Norisoprenoid Responses in Grape Berries to Abscisic Acid and Synthetic Auxin
Source: Int J Mol Sci. 2021 Jan 31;22(3):1420. doi: 10.3390/ijms22031420 (PMC7867017; doi:10.3390/ijms22031420)

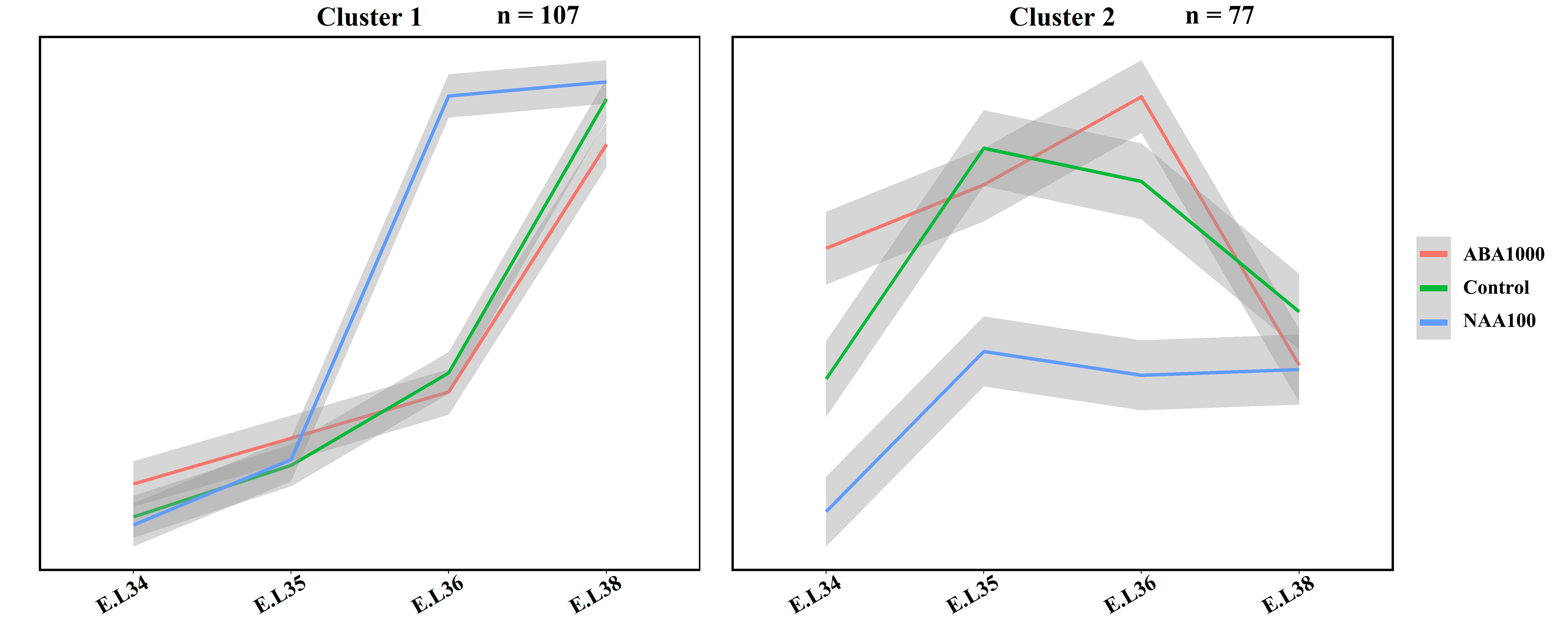

Supplement: Supplementary file 1 [file ijms-22-01420-s001.zip › Supplementary Files/Figure S1.tif]

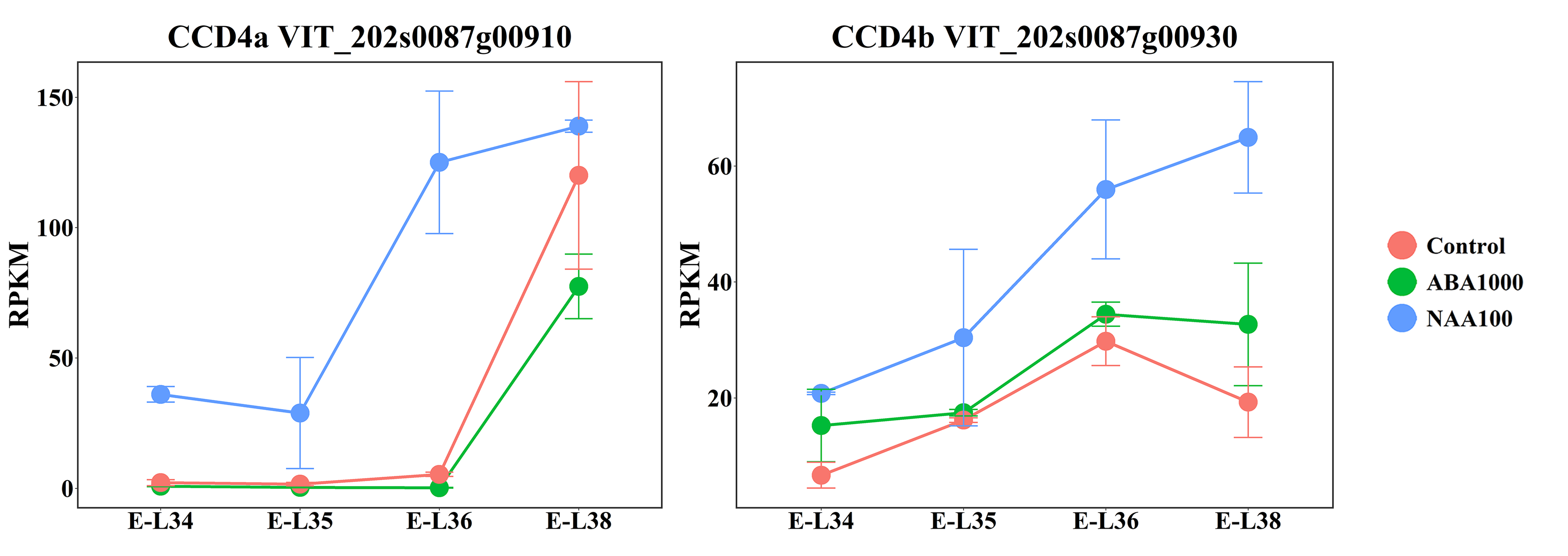

Supplement: Supplementary file 1 [file ijms-22-01420-s001.zip › Supplementary Files/Figure S2.tif]
